# Measurement of Blood-Brain Barrier Permeability with T_1_-Weighted Dynamic Contrast-Enhanced MRI in Brain Tumors: A Comparative Study with Two Different Algorithms

**DOI:** 10.1155/2013/905279

**Published:** 2013-02-20

**Authors:** Maurizio Bergamino, Laura Saitta, Laura Barletta, Laura Bonzano, Giovanni Luigi Mancardi, Lucio Castellan, Jean Louis Ravetti, Luca Roccatagliata

**Affiliations:** ^1^Department of Neuroscience, Rehabilitation, Ophthalmology, Genetics, and Maternal and Child Health, University of Genoa, 16132 Genoa, Italy; ^2^Magnetic Resonance Research Centre on Nervous System Diseases, University of Genoa, 16132 Genoa, Italy; ^3^Department of Diagnostic and Interventional Neuroradiology, San Martino University Hospital, 16132 Genoa, Italy; ^4^Department of Pathology, San Martino University Hospital, 16132 Genoa, Italy; ^5^Department of Health Sciences, University of Genoa, 16132 Genoa, Italy

## Abstract

The purpose of this study was to assess the feasibility of measuring different permeability parameters with T_1_-weighted dynamic contrast-enhanced (DCE) magnetic resonance imaging (MRI) in order to investigate the blood brain-barrier permeability associated with different brain tumors. The Patlak algorithm and the extended Tofts-Kety model were used to this aim. Twenty-five adult patients with tumors of different histological grades were enrolled in this study. MRI examinations were performed at 1.5 T. Multiflip angle, fast low-angle shot, and axial 3D T_1_-weighted images were acquired to calculate T_1_ maps, followed by a DCE acquisition. A region of interest was placed within the tumor of each patient to calculate the mean value of different permeability parameters. Differences in permeability measurements were found between different tumor grades, with higher histological grades characterized by higher permeability values. A significant difference in transfer constant (*K* ^trans^) values was found between the two methods on high-grade tumors; however, both techniques revealed a significant correlation between the histological grade of tumors and their *K* ^trans^ values. Our results suggest that DCE acquisition is feasible in patients with brain tumors and that *K* ^trans^ maps can be easily obtained by these two algorithms, even if the theoretical model adopted could affect the final results.

## 1. Introduction

The blood-brain barrier (BBB) is formed by specialized endothelial cells lining capillaries in the central nervous system (CNS), and it prevents or slows the passage of some drugs and other chemical compounds, radioactive ions, and disease-causing organisms, such as viruses, from the blood into the CNS. BBB breakdown is associated with many CNS-related pathologies, including inflammatory diseases such as multiple sclerosis [1] and chronic and acute cerebrovascular pathology [2, 3]. Pathological modifications of the BBB have also been well described in degenerative diseases such as Alzheimer disease [4]; in addition, it has been shown that in brain tumors the BBB is structurally and functionally abnormal [5]. 

Quantitative investigation of BBB permeability is possible using Magnetic Resonance Imaging (MRI) [5], and it has been applied to the study of brain tumors [6, 7]. In particular, experimental and clinical studies have demonstrated that dynamic contrast-enhanced (DCE) MRI, with a macromolecular contrast agent (CA), can be used to quantify microvascular permeability in tumors [8] and that permeability increases with increasing histological tumor grade [7, 9].

Different theoretical models have been proposed for the analysis of DCE-MRI data in order to find a more accurate approach for the tumor vascular bed and discriminate blood flow and vascular permeability. These models include the standard [10] and the extended Tofts-Kety (ETK) models [11, 12], the adiabatic tissue homogeneity (AATH) model [13, 14], the two-compartment exchange model (2CXM) [15–17], the distributed capillary adiabatic tissue homogeneity (DCATH) model [18], and the gamma capillary transit time model (GCTT) [19]. The absolute quantification of permeability parameters may substantially differ on the basis of the specific model adopted to fit the DCE experimental data [11].

The purpose of this study was to quantify permeability parameters in patients with different histological types of brain tumors by using two different theoretical algorithms. To this aim, we compared the volume transfer constant between blood plasma and the extravascular extracellular space (EES) (*K* ^trans⁡^), the volume of EES per unit volume of tissue (*v*_*e*_) (with 0 ≤ *v*_*e*_ ≤ 1), the vascular volume fraction within the tissue (*v*_*p*_), and the washout rate from the EES back into the blood plasma (*k*_*ep*_). These parameters were calculated using the Patlak algorithm [20], which assumes that *k*_*ep*_ is small, and therefore negligible, and the ETK model. Additionally, the results were evaluated in order to understand if the permeability metrics obtained with the two methods correlated with histological grade.

## 2. Materials and Methods

### 2.1. Patients

Twenty-five adult patients (eight females and seventeen males; mean age ± standard deviation = 54.6 ± 10.0 years; age range = 37–75 years) with different brain tumors were enrolled in this study. The histological grade of each lesion was determined by using the World Health Organization (WHO) classification of brain tumors [21].

### 2.2. MRI Protocol and Image Evaluation

MRI examinations were performed on a 1.5-T clinical system (Siemens Magnetom Avanto, Erlangen, Germany) with an 8-channel head coil, and the following imaging sequences were acquired: T_1_-weighted (TR/TE = 500/10 ms; acquisition matrix = 320 × 384; NEX = 1; field of view = 250 × 300 mm; slice thickness = 5 mm, flip angle = 80°), T_2_-weighted (TR/TE = 3800/95 ms; acquisition matrix = 320 × 384; NEX = 2; field of view = 250 × 300 mm; slice thickness = 5 mm, flip angle = 150°), and fluid attenuated inversion recovery (FLAIR) (TR/TE = 9000/119 ms; acquisition matrix = 320 × 384; NEX = 1; field of view = 250 × 300 mm; slice thickness = 5 mm, flip angle = 150°). Before intravenous injection of the CA, fast low-angle shot (FLASH) axial 3D T_1_-weighted images (TR/TE = 6.7/1.0 ms; acquisition matrix = 320 × 384; NEX = 1; field of view = 250 × 300 mm; slice thickness = 5 mm) were acquired with multiple flip angles (5°, 10°, 15°, 20°, and 30°).

Acquisition of a DCE-MRI sequence was started immediately after intravenous administration of a gadolinium-based CA (gadobutrol 0.1 ml/kg, Gadovist) by a power injector (Spectris Solaris EP Medrad) at a rate of 5 mL/s. Dynamic axial 3D T_1_-weighted TurboFLASH images (TR/TE = 6.7/1.0 ms; acquisition matrix = 320 × 384; NEX = 1; field of view = 250 × 300 mm; slice thickness = 5 mm; flip angle = 30°) were acquired for 30 time points.

Permeability maps were created as described in the Theory and Data Analysis section, and the mean of *K* ^trans⁡^, *v*_*e*_, and *v*_*p*_ was obtained for each patient in a region of interest (ROI) within the tumor. 

### 2.3. The Extended Tofts-Kety Model

The Patlak algorithm and the ETK model were used in order to calculate different permeability parameters.

The tracer concentration in tissue, *C*_*t*_, and the tracer concentration in arterial blood plasma, *C*_*p*_, are related by the differential equation

(1) $$\begin{array}{c} \frac{dC_{t}}{dt}=K^{\mathrm{trans}}C_{p}-k_{ep}C_{t}, \end{array}$$

where *k*_*ep*_ = *K* ^trans⁡^/*v*_*e*_ is the rate constant [10].

The solution of (1), using the initial conditions *C*_*p*_ = *C*_*t*_ = 0 at *t* = 0, is

(2) $$\begin{array}{c} C_{t}\left(t\right)=K^{\mathrm{trans}}\int C_{p}\left(\tau \right)\exp\left[-k_{ep}\left(t-\tau \right)\right]d\tau , \end{array}$$

where *t* represents the current time step, *τ* is the variable of integration, *C*_*t*_(*t*) is the time course of the CA concentration in the tissue compartment, and *C*_*p*_(*t*) is the time course of the CA concentration in the plasma (AIF) [22, 23]. 

Equation (2) is the standard Tofts-Kety model and is acceptable in tumors with no large increase in blood volume; however, it is not valid in other contexts, for instance when blood volume can increase markedly in neoplasms [24]. Models of additional sophistication are required to adequately describe these cases. It is possible to extend (2) to include the concentration of CA in the blood plasma:

(3) $$\begin{array}{c} C_{t}\left(t\right)=K^{\mathrm{trans}}\int C_{p}\left(\tau \right)\exp\left[-k_{ep}\left(t-\tau \right)\right]d\tau +v_{p}C_{p}\left(t\right). \end{array}$$

Equation (3) is the ETK model, where *v*_*p*_ is the vascular volume fraction within the tissue. Equations (2) and (3) are the basis of the most of DCE-MRI experiments currently being reported in the literature. The general approach is to measure *C*_*p*_ and *C*_*t*_ time courses and perform a nonlinear least squares fit of these equations to such data. By varying the parameters in those equations, it is possible to obtain estimates on *K* ^trans⁡^, *v*_*e*_ and *v*_*p*_. For this study, postprocessing analysis for the ETK model was performed using in-house software.

### 2.4. Patlak Analysis

This analysis assumes that the rate constant between EES and blood plasma (*k*_*ep*_) in (3) can be ignored due to low permeability and short measuring time. In these cases, (3) can be reduced to

(4) $$\begin{array}{c} C_{t}\left(t\right)=v_{p}C_{p}\left(\tau \right)+K^{\mathrm{trans}}\int C_{p}\left(\tau \right)d\tau . \end{array}$$

Using the Patlak analysis, it is possible to linearize (4) to obtain a graph of the ratio *C*_*t*_(*t*)/*C*_*p*_(*t*) versus ∫*C*_*p*_(*τ*)*dτ*/*C*_*p*_(*t*) in order to calculate the values of *K* ^trans⁡^ and *v*_*p*_. It is useful to keep in mind that this model does not take into account the backflow and, therefore, the results could have some limitations [25]. For this work, postprocessing analysis for the Patlak algorithm was performed using TOPPCAT (T-One weighted Perfusion imaging Parameter Calculation Toolkit) [https://dblab.duhs.duke.edu/] [26], which is a free permeability analysis software available on Internet.

### 2.5. The Voxel Relation Rate

The calculation of the permeability parameters by using (2), (3), or (4) requires the values of *S*_0_, the equilibrium longitudinal magnetization, and *T*_1_ pregadolinium mapping for each voxel. Precontrast *T*_1_ mapping of tissue can be obtained with different approaches; a common method employs multiple 3D gradient echo acquisitions at multiple variable flip angles. For this purpose, pulse sequence and flip angle must be chosen to maximize the sampling rate within the constraint of an adequate signal-to-noise ratio (SNR). Another frequently used method to calculate the *T*_1_ baseline values utilizes 2D T_1_-weighted inversion recovery scans at various inversion times [27, 28]. This method is considered more accurate than the variable flip angle method, but it is usually more time consuming. 

In our study, we chose to use acquisitions at multiple variable flip angles to obtain *S*_0_ and *T*_1_ values. In FLASH MRI with a complete spoiling of the transverse magnetization, the steady-state signal is given by

(5) $$\begin{array}{c} S_{\alpha }=\frac{S_{0}\left(1-\exp\left[-\text{TR}/T_{1}\right]\right)\sin\alpha }{1-\cos\;\alpha \cdot \exp\left[-\text{TR}/T_{1}\right]}, \end{array}$$

where TR is the repetition time and *α* is the flip angle for TR >> T2* [29]. To construct the *T*_1_ and *S*_0_ maps, it is necessary to fit the data from FLASH sequence by a linearization of (5). The *T*_1_ and *S*_0_ values are then utilized to estimate the voxel *R*_1_ time courses, or relaxation rates, from the acquired signal intensity time courses,

(6) R1(t) =−1TR  ×ln⁡[(1−(S(t)−S(0)S0sinα+1−m1−(m·cos⁡α)))   ×(1−cos⁡α(S(t)−S(0)S0sinα+(1−m1−(m·cos⁡α))))−1],

where *m* = exp⁡[−TR/*T*_1_], *α* is the flip angle of the DCE-MR sequence, and *S*(0) and *S*(*t*) are the signal intensities at time *t* = 0 and time *t,* respectively. The relaxation rate *R*_1_(*t*) is related to the tracer concentration *C*_*t*_(*t*) by

(7) $$\begin{array}{c} R_{1}\left(t\right)=R_{10}+r_{1}C\left(t\right), \end{array}$$

where *R*_10_ is the relaxation rate before tracer injection and *r*_1_ is the relaxivity of the CA, which varies with the molecule's contrast type (in our case *r*_1_ = 4.3 mM^−1^s^−1^) [30].

### 2.6. Statistical Analysis

The Kolmogorov-Smirnov test of normality was used to determine whether the distribution of values was normal, and comparisons between these two theoretical models were performed using the student's *t*-test. A *P* value lower than 0.05 was considered to indicate statistical significance for all comparisons. The relationship between *K* ^trans⁡^ and tumor grade was assessed by using Pearson correlation coefficients.

## 3. Results

The study group consisted of twenty-five patients, whose histological diagnoses were as follows seventeen glioblastoma multiforme (WHO grade IV), two oligoastrocytoma (WHO grade III), two anaplastic astrocytomas (WHO grade III), one pleomorphic xanthoastrocytoma (WHO grade III), and three astrocytomas (WHO grade II). 

**Figure 1:**
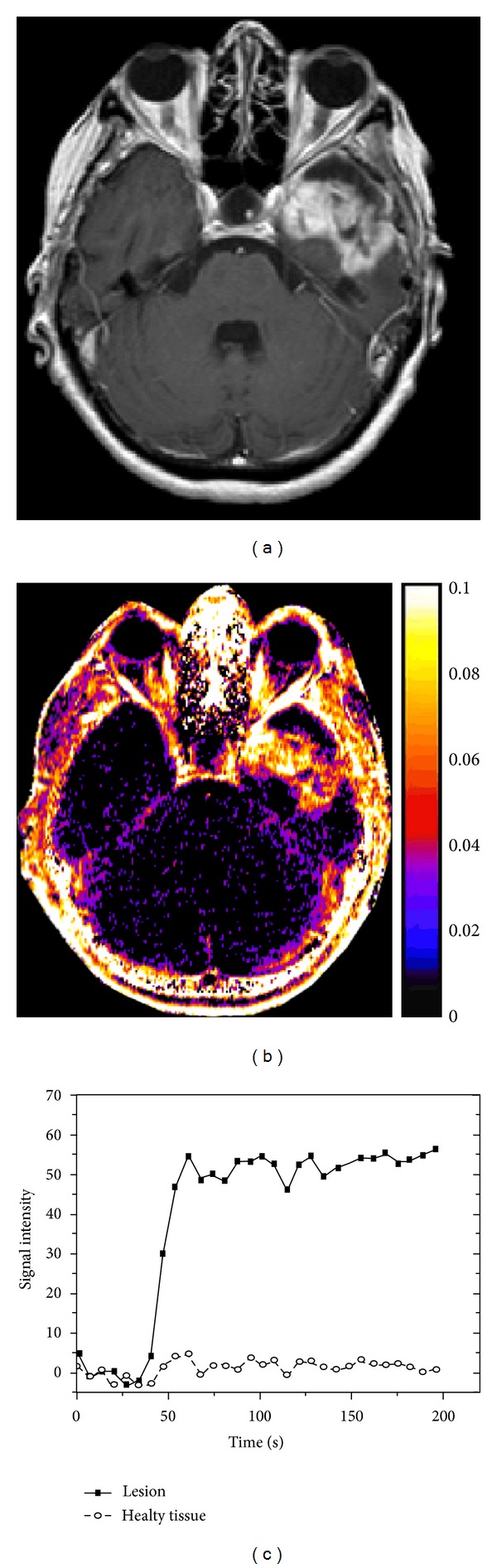
Image and intensity data from a 69-year old female patient with glioblastoma multiforme (WHO IV). (a) T_1_-weighted postcontrast image. (b) Relative *K* ^trans⁡^ map. (c) Signal intensity plot for a region of the tumor and for a portion of healthy brain tissue.

Differences in permeability measurements were found between different tumor grades, with higher histological grades characterized by higher permeability values. In particular, for grade IV tumors, the mean *K* ^trans⁡^ value calculated by using the Patlak algorithm was 0.039 ± 0.016 min^−1^, and the mean *K* ^trans⁡^ value calculated by using the ETK model was 0.051 ± 0.015 min^−1^ (Figure 1). For grade III tumors, the mean *K* ^trans⁡^ value was 0.032 ± 0.011 min^−1^ for the Patlak algorithm and 0.043 ± 0.022 min^−1^ for the ETK model. In grade II tumors, we obtained a mean *K* ^trans⁡^ value of 0.010 ± 0.006 min^−1^ with the Patlak algorithm and a mean *K* ^trans⁡^ value of 0.011 ± 0.007 min^−1^ with the ETK model. Statistical analysis showed significant differences in the mean *K* ^trans⁡^ values obtained with the Patlak algorithm versus those obtained with the ETK model for grade IV tumors (*P* = 0.049, *t* = 2.04) and for high grade tumors (i.e., WHO IV and WHO III grouped together) (*P* = 0.031, *t* = 2.02). No significant differences were found between mean *K* ^trans⁡^ values obtained with the two algorithms for grade II tumors. In addition, we did not find significant differences in mean *v*_*p*_ values between the two methods in both high-grade tumors and low-grade tumors.

Additionally, we calculated *v*_*e*_ only for the ETK model, finding for high-grade tumors (WHO IV and WHO III grouped together) a mean *v*_*e*_ of 0.15 ± 0.05, whilst a mean *v*_*e*_ of 0.017 ± 0.006 was found for low-grade tumors.

**Figure 2:**
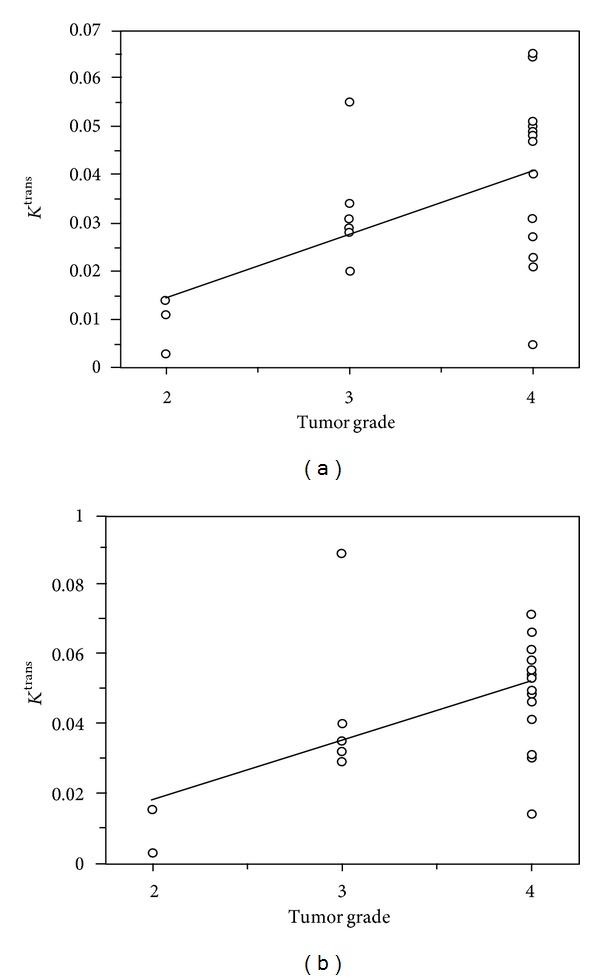
A statistically significant correlation was observed between mean *K* ^trans⁡^ and tumor grade for both Patlak algorithm ((a): *r* = 0.54  (*P* = 0.004)) and for the ETK model ((b): *r* = 0.58  (*P* = 0.002)).

The histological grade of tumors for these patients was found to have a significant correlation with *K* ^trans⁡^ values. For the Patlak algorithm, we found a Pearson correlation coefficient *r* = 0.54  (*P* = 0.004), and for the ETK model we found a Pearson correlation coefficient = 0.58(*P* = 0.002). Correlations between tumor grade and mean *K* ^trans⁡^ values are shown in Figure 2. No significant correlation was found between *v*_*p*_ and tumor grade for both methods. 

## 4. Discussion

Over the past two decades, important progress has been made in the development of a robust method to noninvasively quantify the microvascular permeability of the BBB for clinical use [11]. BBB can be altered in brain tumors neoangiogenesis because of new vessels, which are structurally and functionally abnormal. This abnormality impairs effective delivery of therapeutic agents to all regions of tumors, creates an abnormal microenvironment (e.g., hypoxia) that reduces the effectiveness of radiation and chemotherapy, and selects for more malignant cells [31]. BBB disruption caused by tumors is heterogeneous, thus, the permeability can vary widely in different areas of the same tumor. Creation of parametric maps with a *K* ^trans⁡^ value for each voxel can be of practical importance and useful, for instance, to guide the biopsy target [32].

Different theoretical models can be used to fit DCE-MRI experimental data, and the resulting permeability parameters can be influenced by the model used. In this study, 25 patients with different brain tumors underwent DCE MRI. The Patlak algorithm, which assumes that the washout rate from the EES back into the blood plasma is small and therefore negligible, and the ETK model were applied to estimate kinetic parameters. Our results showed that *K* ^trans⁡^ mean values in ROIs within high-grade tumors were significantly different between the two methodologies, with higher *K* ^trans⁡^ mean values calculated with the ETK model. 

In many studies, *K* ^trans⁡^ is estimated by applying the TK model or the ETK model. However, the main disadvantage of the TK model is that it overestimates *K* ^trans⁡^ in highly vascularized regions, since the contribution of intravascular CA to the signal enhancement is mistaken as tracer that enters the EES and thus appears to reflect permeability [33]. Harrer et al. [33] used the TK and ETK models to estimate permeability on 18 high-grade gliomas, and they found that *K* ^trans⁡^ values calculated by using the TK model were considerably higher than the *K* ^trans⁡^ values obtained using the ETK technique. On the other hand, Port et al. [34] estimated the difference between noncompartmental, TK and ETK models on 20 patients with recurrent glioblastoma, finding significant differences in the results obtained with the different models.

 In our study, we found statistically different *K* ^trans⁡^ values for high-grade tumors, but no significant difference for low-grade tumors. This result may be related to the low number of patients with grade II tumors in this study, but biological differences between high-grade tumors and low-grade tumors can also be associated with these findings.

Both techniques reveal a significant correlation between the histological grade of tumors and their *K* ^trans⁡^ values. These data are in line with other results that can be found in the literature [35, 36].

## 5. Conclusion

In conclusion, we demonstrated that DCE acquisition is feasible in patients with brain tumors and that the choice of the postprocessing tool can influence the permeability metrics. In particular, the use of the Patlak algorithm versus the ETK model can lead to statistically significant differences in the *K* ^trans⁡^ values. In our small sample, this difference in results did not affect the correlation with histological grade.
